# Asymptomatic Necrotizing Granulomatous Disease of the Neck With Unknown Etiology

**DOI:** 10.7759/cureus.37010

**Published:** 2023-04-01

**Authors:** Emily S Sagalow, William Montagne, Nathan Lloyd, Shadaba Asad, Robert C Wang

**Affiliations:** 1 Otolaryngology-Head and Neck Surgery, University of Nevada Las Vegas (UNLV) School of Medicine, Las Vegas, USA; 2 Infectious Disease, University of Nevada Las Vegas (UNLV) School of Medicine, Las Vegas, USA

**Keywords:** otolaryngology, infectious disease, neck mass, necrotizing granulomatous disease, lymphadenitis, chat gpt

## Abstract

Patients presenting with unilateral neck masses is not an uncommon occurrence in an otolaryngology clinic. Especially those with risk factors such as older age and a history of smoking or drinking along with certain characteristics of the mass including rapid growth, immobility, and the presence of other masses elsewhere in the head and neck that can lead to more concerning etiologies such as cancer. However, in those who are younger with non-tender unilateral mobile masses, the differential is wide. We present the case of a 30-year-old male who presented with a non-tender left-sided neck mass with no associated or systemic symptoms. Workup including labs for HIV, syphilis, and fungal stains was negative. Pathology demonstrated lymphadenitis with necrotizing granulomas with no recurrence of symptoms after excisional biopsy. The patient continued to have no associated symptoms or recurrent mass thus no further workup was deemed necessary. Although unilateral neck mass and lymphadenitis with necrotizing lymphadenitis have a broad differential diagnosis, this patient’s etiology continues to be unknown.

## Introduction

Patients with a unilateral neck mass of variable size, length of time, and etiology often present at an otolaryngology clinic. For those with risk factors such as older age or significant smoking or drinking history combined with characteristics of the mass such as immobility, large size, invasion into adjacent structures, rapid growth, and other masses elsewhere in the head and neck can lead healthcare providers to prioritize more concerning etiologies such as malignancy. On the other hand, in younger patients with unilateral mobile masses, the differential is wider and malignancy may rank lower on the list of etiologies. This differential includes lymphadenopathy due to infection, autoimmune disorder, or malignancy, thyroid nodule, salivary gland tumor, lipoma, branchial cleft cyst, carotid body tumor, metastatic cancer, and soft tissue sarcoma [1-9].

We present a peculiar case of a 30-year-old male who presented to the otolaryngology clinic with a unilateral neck mass with pathology demonstrating lymphadenitis with necrotizing granulomas, negative workup leading to unknown etiology, and spontaneous resolution of symptoms after excisional biopsy.

This article was written using the assistance of the AI platform ChatGPT. The corresponding search terms used to assist in writing this article are demonstrated in the Appendices.

## Case presentation

A 30-year-old male with no significant past medical history presented to the otolaryngology clinic in March 2021 after incidentally noticing a non-tender lump on the left side of his neck without any other associated symptoms. At the time, a review of systems was negative for dysphagia, sore throat, hemoptysis, weight loss, anorexia, fever, chills, rigors, redness, swelling, pain, or any drainage from the mass. He denied smoking history, warts, allergies, recent travel, history of incarceration, IV drug use, homelessness or contact with anyone with known tuberculosis (TB), ownership of other pets aside from two cats, and herbal or nutritional supplements other than fish oil capsules, vitamin-D, and protein powder.

**Figure 1:**
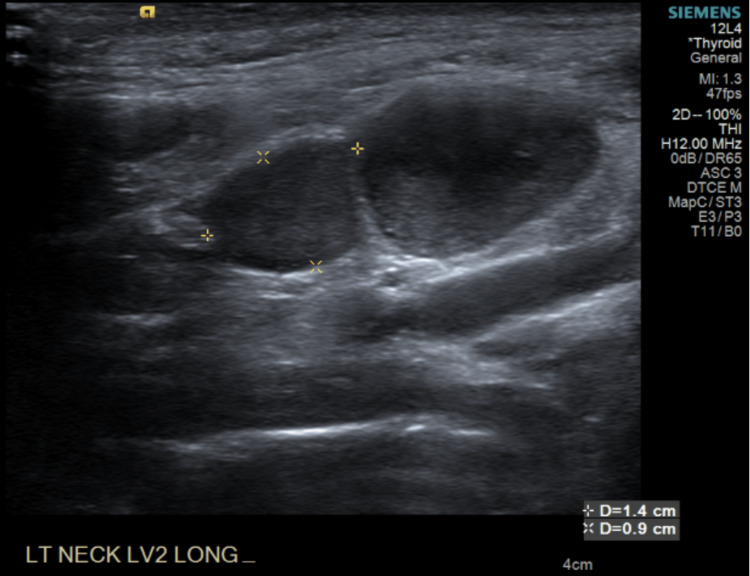
Initial neck ultrasound This ultrasound demonstrated hypoechoic circumscribed nodular structures in the left neck at level 2, the largest of which measured up to 1.9 x 8 2.1 x 1.3 cm. There was no normal fatty hilum seen.

**Figure 2:**
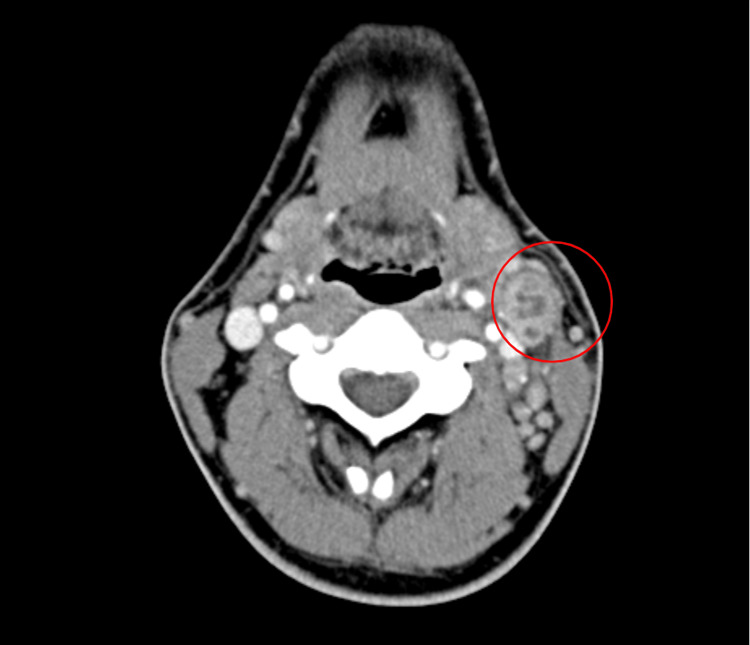
Initial neck axial view computed tomography scan with and without contrast The red circle demonstrates the mass of interest

**Figure 3:**
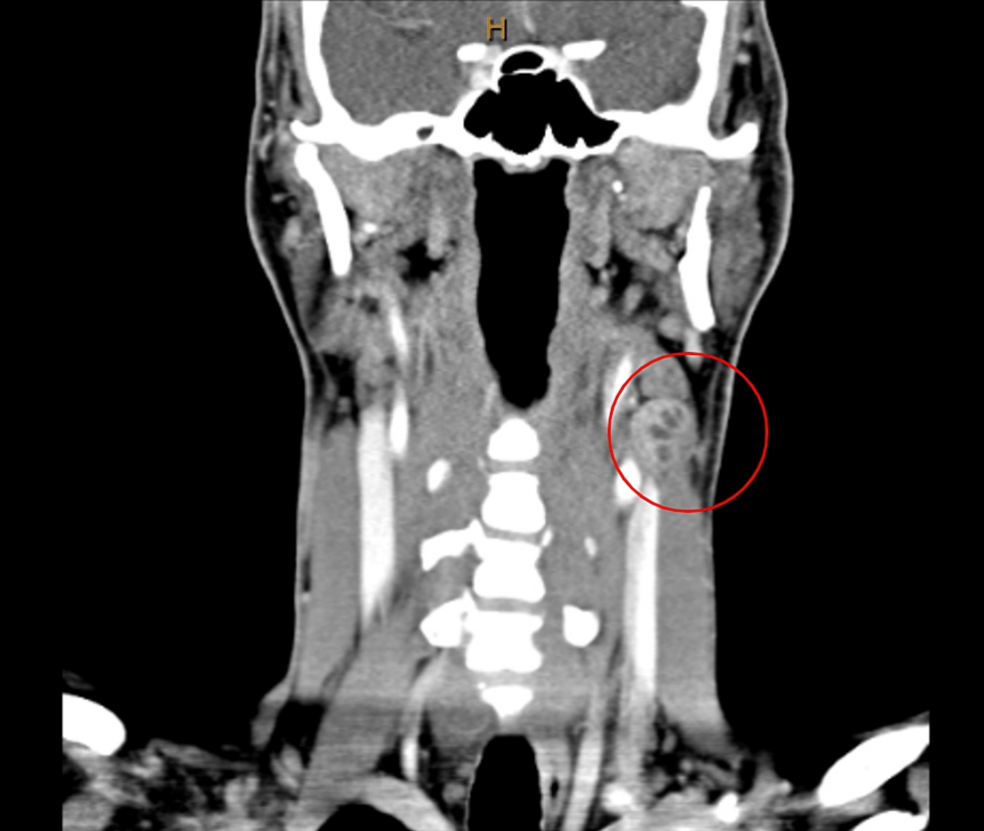
Initial neck coronal view computed tomography scan with and without contrast The red circle demonstrates the mass of interest

Workup included neck ultrasound (US) that revealed multiple left-sided hypoechoic circumscribed nodular structures the largest of which measured up to 1.9 x 8 2.1 x 1.3 cm without normal fatty hilum. The neck CT demonstrated a mass posterior to the left submandibular gland and anterior to the sternocleidomastoid muscle and carotid vasculature, directly abutting the anterolateral left jugular vein measuring 2.1 cm x 1.8 cm (Figures 1-3).

Augmentin 875 mg twice a day for two weeks was prescribed. The mass did not resolve after one month so the patient underwent US fine needle aspiration (FNA) of the neck mass that demonstrated lymphadenitis. Positron emission tomography (PET) CT was performed that demonstrated increased uptake of 2 foci of uptake SUV 17-21, 1.4 and 2.4 cm at left level 2A with no asymmetrical uptake in the oropharynx.

The patient was then referred to the infectious disease clinic. At that time, further testing including HIV, syphilis screen, fungal studies including Coccidioidomycosis serologies, erythrocyte sedimentation rate (ESR), and C-reactive protein (CRP) was unremarkable. 

In June 2021, the patient underwent panendoscopy with an excisional biopsy of the left level 2A lymph node. The patient was recommended to undergo modified radical neck dissection with tonsillar and/or base of tongue resection with transoral robotic surgery and/or carbon dioxide (CO2) laser if pathology demonstrated squamous cell carcinoma as the primary etiology was still unknown at this point. 

Histopathology was consistent with necrotizing granulomas. Periodic acid-Schiff (PAS) and acid-fast bacilli (AFB) stains were both negative. Cultures including fungal and AFB cultures remained with no growth. Tuberculin skin test purified protein derivative (PPD) was placed with a subsequent negative reading. 

The patient returned to the clinic with concerns about a new palpable right-sided neck lymph node. Neck US at this time demonstrated an enlarged hypoechoic lymph node without fatty hilum in right level 5. Follow-up neck CT demonstrated a 1.1 cm x 0.6 cm of the right posterior neck triangle.

Extensive workup remained unrevealing and the patient remained asymptomatic. Repeat neck US six and 18 months later were negative for adenopathy. Pertinent physical exam findings at the most recent follow-up 35 months after the initial presentation included a well-headed excisional biopsy scar of the left neck with an overall normal neck appearance and symmetry with no masses palpated. At this point, no further workup was recommended as the patient remained asymptomatic.

## Discussion

There are many potential causes of a unilateral neck mass in a 30-year-old male. The differential diagnosis would depend on the characteristics of the mass, such as its size, location, and associated symptoms as well as the medical history and physical examination findings of the individual. Some possible causes of a unilateral neck mass in a 30-year-old male could include lymphadenopathy (due to infection, autoimmune disorder, or malignancy), thyroid nodule (benign or malignant), salivary gland tumor, lipoma (noncancerous tumor made up of fat cells that can occur anywhere on the body, but are usually soft, mobile, and painless), branchial cleft cyst (a congenital cystic mass that arises from remnants of the branchial cleft during embryonic development), carotid body tumor (a benign tumor that arises from cells in the carotid artery that can cause a pulsatile neck mass), metastatic cancer, and soft tissue sarcoma (a rare type of cancer that can arise in the muscles, fat, or other soft tissues of the neck) [1-9].

Acute lymphadenitis is inflammation and enlargement of the lymph nodes that usually occurs due to an infection. The differential diagnosis for acute lymphadenitis includes bacterial infections (such as staphylococcal infections, cat-scratch fever, and TB), viral infections (mononucleosis, measles, rubella, and HIV), fungal infections (histoplasmosis and coccidioidomycosis), parasitic infections (toxoplasmosis, leishmaniasis, and filariasis), autoimmune disorders (systemic lupus erythematosus (SLE) and rheumatoid arthritis), malignancy (lymphoma and metastatic cancers), and reactive lymphadenopathy (response to a variety of stimuli such as drug hypersensitivity, immunization, and lymphoproliferative disorders) [10-18].

Lymphadenitis with necrotizing granulomas has several etiologies. Tuberculosis is a bacterial infection that can affect multiple organs, including the lymph nodes with TB lymphadenitis being a common form of extrapulmonary TB that can cause necrotizing granulomas. Certain fungal infections such as histoplasmosis, coccidioidomycosis, blastomycosis, and cryptococcosis can cause necrotizing granulomas in lymph nodes. Cat-scratch disease, a bacterial infection that occurs after a scratch or bites from a cat infected with *Bartonella henselae*, typically causes lymphadenitis with regional lymph node enlargement and necrotizing granulomas. Sarcoidosis, a systemic disease that can affect multiple organs including the lymph nodes, can cause granulomas that resemble TB but are not caused by an infection. Kikuchi-Fujimoto disease, a rare benign condition, can cause necrotizing lymphadenitis, particularly in young Asian women [15-16]. Some types of lymphoma, such as Hodgkin lymphoma or anaplastic large cell lymphoma, can present with necrotizing granulomas in lymph nodes. Melioidosis, a bacterial infection caused by *Burkholderia pseudomallei *that is endemic in certain regions, can cause necrotizing granulomas in lymph nodes and other organs. In some cases, necrotizing granulomas can be caused by non-infectious conditions such as granulomatosis with polyangiitis or rheumatoid arthritis [19-20].

Our patient underwent extensive workup through the otolaryngology and infectious disease departments along with testing for HIV, syphilis, and fungal studies including coccidioidomycosis serologies, ESR, CRP, PAS and AFB stains on the excisional biopsy specimen, fungal and AFB cultures, and PPD that were all unremarkable. The patient had an eventual resolution of the neck mass spontaneously without recurrence. He did not experience any other associated or systemic symptoms throughout this disease course.

## Conclusions

In this report, we describe a 30-year-old male who presented with an asymptomatic non-tender left-sided neck mass. Workup including viral and bacterial labs and fungal stains was negative. Excisional biopsy pathology demonstrated lymphadenitis with necrotizing granulomas. The patient experienced no recurrence of neck mass or associated symptoms post-excision thus no further workup was deemed necessary by both the otolaryngology and infectious disease medical teams collaboratively treating the patient. Although the differential diagnoses for both unilateral neck masses and lymphadenitis with necrotizing granulomata are broad, this patient’s etiology continues to be unknown.

## Figures and Tables

**Figure 4:**
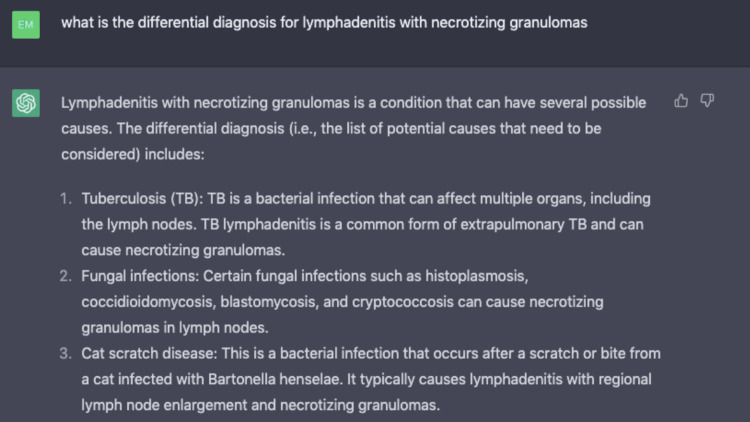
Screenshot of ChatGPT search phrase 1 Pictured here is the ChatGPT response to the search phrase "what is the differential diagnosis for lymphadenitis with necrotizing granulomas"

**Figure 5:**
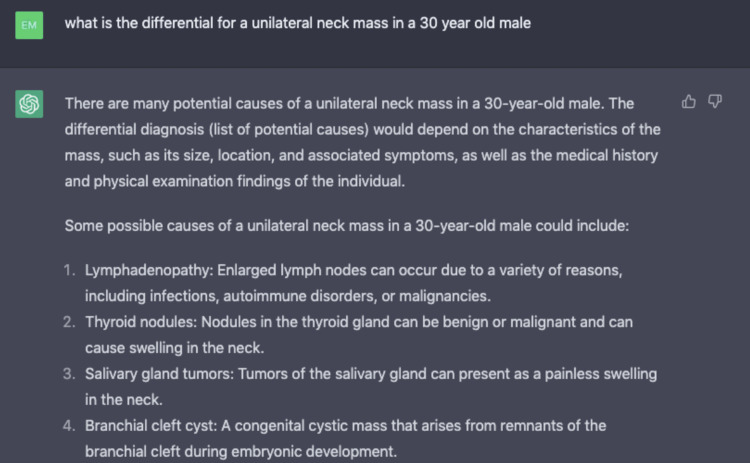
Screenshot of ChatGPT search phrase 2 Pictured here is the ChatGPT response to the search phrase "what is the differential for a unilateral neck mass in a 30-year-old male"
